# Supplementary material for: Caveolin-1 temporal modulation enhances antibody drug efficacy in heterogeneous gastric cancer
Source: Nat Commun. 2022 May 9;13:2526. doi: 10.1038/s41467-022-30142-9 (PMC9085816; doi:10.1038/s41467-022-30142-9)
Supplement: Supplementary file 2 — Reporting Summary [file 41467_2022_30142_MOESM2_ESM.pdf]

nature portfolio

Corresponding author(s): Patricia M.R. Pereira, PhD
Jason S. Lewis, PhD

Last updated by author(s): Mar 22, 2022

Reporting Summary

Nature Portfolio wishes to improve the reproducibility of the work that we publish. This form provides structure for consistency and transparency in reporting. For further information on Nature Portfolio policies, see our Editorial Policies and the Editorial Policy Checklist.

Statistics

For all statistical analyses, confirm that the following items are present in the figure legend, table legend, main text, or Methods section.

n/a	Confirmed	
☐	☒	The exact sample size (*n*) for each experimental group/condition, given as a discrete number and unit of measurement
☐	☒	A statement on whether measurements were taken from distinct samples or whether the same sample was measured repeatedly
☐	☒	The statistical test(s) used AND whether they are one- or two-sided<br>*Only common tests should be described solely by name; describe more complex techniques in the Methods section.*
☒	☐	A description of all covariates tested
☒	☐	A description of any assumptions or corrections, such as tests of normality and adjustment for multiple comparisons
☐	☒	A full description of the statistical parameters including central tendency (e.g. means) or other basic estimates (e.g. regression coefficient) AND variation (e.g. standard deviation) or associated estimates of uncertainty (e.g. confidence intervals)
☐	☒	For null hypothesis testing, the test statistic (e.g. *F*, *t*, *r*) with confidence intervals, effect sizes, degrees of freedom and *P* value noted<br>*Give P values as exact values whenever suitable.*
☒	☐	For Bayesian analysis, information on the choice of priors and Markov chain Monte Carlo settings
☒	☐	For hierarchical and complex designs, identification of the appropriate level for tests and full reporting of outcomes
☒	☐	Estimates of effect sizes (e.g. Cohen's *d*, Pearson's *r*), indicating how they were calculated

Our web collection on statistics for biologists contains articles on many of the points above.

Software and code

Policy information about availability of computer code

Data collection	No software was used for data collection.
Data analysis	Membranes analyzed using ImageJ/FIJI (NIH, USA) to measure fluorescence intensity after applying a median filter and background subtraction.<br><br>FACs data were analyzed with Flowjo software (Flowjo LLC v10.7.1). Flow cytometry was performed in the MACSQuant Analyzer 10.<br><br>PET images were analyzed using ASIPro VM software (Concorde Microsystems).

For manuscripts utilizing custom algorithms or software that are central to the research but not yet described in published literature, software must be made available to editors and reviewers. We strongly encourage code deposition in a community repository (e.g. GitHub). See the Nature Portfolio guidelines for submitting code & software for further information.

Data

Policy information about availability of data

All manuscripts must include a data availability statement. This statement should provide the following information, where applicable:

- Accession codes, unique identifiers, or web links for publicly available datasets
- A description of any restrictions on data availability
- For clinical datasets or third party data, please ensure that the statement adheres to our policy

All data generated or analyzed during this study are included in this published article (and its supplementary information files). Source data are provided with this paper.

Field-specific reporting

Please select the one below that is the best fit for your research. If you are not sure, read the appropriate sections before making your selection.

☒ Life sciences ☐ Behavioural & social sciences ☐ Ecological, evolutionary & environmental sciences

For a reference copy of the document with all sections, see nature.com/documents/nr-reporting-summary-flat.pdf

Life sciences study design

All studies must disclose on these points even when the disclosure is negative.

Sample size

Cohorts of mice were always n = 5 for imaging and n = 10 for therapy. The number of mice used in our experiments was defined according to institutional guidelines and animal protocols.

Statistical analyses performed to determine sample size:
Biodistribution kinetics will be quantified using SUV, volume of distribution, uptake, and clearance half-times. The level of biodistribution will be analyzed using a linear regression, with the treatment as covariate adjusted on the time point. In a second model, an interaction between treatment and time will be added. If the interaction is significant, the biodistribution will be compared separately at each time point. To conclude that antibody tumor uptake is 50% higher in the statin group than in the control (power of 80%, 2-sided alpha of 0.05) we will need 5 mice/cohort per biodistribution time point. For the therapeutic studies, the Area Under the tumor growth Curve (AUC) will be compared using the Vardi test. To show that the tumor size is smaller in the antibody/caveolae-modulator group as compared to the antibody-only group, 10 mice per group are needed to reach a power of 80% with a 1-sided alpha of 0.05. Statistically significant differences between mean values will be determined using analysis of variances (ANOVA) coupled to Scheffé's method or (for classification) a Student’s t-test using R statistical computing. Differences at the 95% confidence level ($p < 0.05$) will be considered significant.
For in vitro studies, sample sizes of 3 independent
experiments (3 per group) will achieve 97.0% power to test the difference of number of viable cells between two
groups at a significance level of 95% using a two-sided two-sample unequal-variance t-test.

Data exclusions

No data were excluded from the analyses.

Replication

All attempts at replication were successful. n=3 independent experiments.

Randomization

When the volume of xenografts reached approximately 100 mm3, mice were randomized into groups and treatments initiated.

Blinding

Personal performing the therapeutic experiments were blinded regarding the treatments.
Researchers were blinded to in vitro cell treatments, during data collection; as well as during staining of CAV1 and HER2 in the different tumor samples and in vitro samples.

Reporting for specific materials, systems and methods

We require information from authors about some types of materials, experimental systems and methods used in many studies. Here, indicate whether each material, system or method listed is relevant to your study. If you are not sure if a list item applies to your research, read the appropriate section before selecting a response.

Materials & experimental systems

n/a	Involved in the study	
☐	☒	Antibodies
☐	☒	Eukaryotic cell lines
☒	☐	Palaeontology and archaeology
☐	☒	Animals and other organisms
☒	☐	Human research participants
☐	☒	Clinical data
☐	☐	Dual use research of concern

Methods

n/a	Involved in the study	
☒	☐	ChIP-seq
☒	☐	Flow cytometry
☒	☐	MRI-based neuroimaging

Antibodies

Antibodies used

Anti-HER2 antibody Herceptin (obtained from the MSK Hospital Pharmacy).

Anti-HER2 antibody-drug conjugate T-DM1 (obtained from the MSK Hospital Pharmacy).

Antibodies used for WB analysis: Crabbit anti-CAV1 1:500 (Abcam, ab2910), rabbit anti-HER2 1:800 (Abcam, ab131490), mouse anti β-actin 1:20,000 (Sigma, A1978), rabbit anti-ubiquitin 1:1,000 (Cell Signaling Technology, 3933S), mouse anti-ERK 1:100 (Invitrogen, 14-9108-80), rabbit anti-pERK 1:500 (Invitrogen, 700012), rabbit anti-AKT 1:1,000 (Cell Signaling Technology, 9272S), rabbit anti-pAKT, 1:2,000 (Cell Signaling Technology, 4060S), rabbit anti-cleaved PARP, 1:1,000 (Cell Signaling Technology, 9541S), rabbit anti-

	pHER2, 1:500 (Abcam, ab53290), rabbit anti-HER3, 1:500 (Abcam, ab32121), rabbit anti-pHER3, 1:2,500 (Abcam, ab76469), rabbit anti-EGFR 1:1,000 (Abcam, ab52894), rabbit anti-pEGFR 1:500 (Abcam, ab40815), mouse anti-pTyr 0.5 µg/mL (EMD Millipore, 05-321X), rabbit anti-CREB 1:1,000 (Cell Signaling Technology, 9197S), rabbit anti-pCREB 1:1,000 (Cell Signaling Technology, 9198S). IRDye®800CW anti-rabbit or anti-mouse IgG 1:15,000 (LI-COR Biosciences).<br><br>The MAPK Phosphorylation Antibody Array (Abcam, ab211061, 1:200) was used to determine MAPK signaling changes.<br><br>The rabbit anti-LAMP-1 primary antibody (ab24170, Abcam) was used for immunofluorescence.
Validation	Clinical-grade trastuzumab (Herceptin TM, Genentech, South San Francisco, CA) or ado-trastuzumab emtansine (T-DM1, Genentech/ Roche) was used in the experiments.<br>doi: 10.1097/RLU.0000000000001820<br>doi: 10.1158/2159-8290.CD-20-0215.<br><br>Antibodies for Western blot were validated in cancer cell lines expressing varying levels of the target of interest. Antibody validation against the respective specie was noted in the datasheet and online databases of the manufacturer.

Eukaryotic cell lines

Policy information about cell lines

Cell line source(s)	Human gastric cancer (GC) cell lines NCIN87, AGS, SNU5, SNU1, and KATOIII, were purchased from American Type Culture Collection (ATCC). MKN45 (GmbH) gastric cancer cells, embryonic kidney 293 cells (HEK 293; ATCC), and 293FT (Thermo Fisher Scientific) packaging cells were gifts from the Rudin Lab and Weisser Lab at MSK.<br><br>An 81 mL leukapheresis pack containing 5.50 × 109 white cells with a viability of 98% was obtained from STEMCELL Technologies
Authentication	All the cell lines were authenticated at Memorial Sloan-Kettering Cancer Center (MSKCC) integrated genomics operation core using short tandem repeat analysis.
Mycoplasma contamination	All cell lines tested negative for mycoplasma contamination.
Commonly misidentified lines<br>(See ICLAC register)	This study did not use commonly misidentified lines.

Animals and other organisms

Policy information about studies involving animals; ARRIVE guidelines recommended for reporting animal research

Laboratory animals	Female athymic nude mice nu/nu (8 to 10 weeks old) were obtained from Charles River Laboratories.<br><br>The experimentation involving animals followed the guidelines approved by the Research Animal Resource Center and Institutional Animal Care and Use Committee at MSK (New York, NY), the ARRIVE guidelines, and the guidelines for the welfare and use of animals in cancer research. The mice were housed in type II polycarbonate cages, fed with sterilized standard laboratory diet and received sterile water ad libitum. The animals were housed at approximately 22 °C, 60% relative humidity, and a 12 h light, 12 h dark cycle was maintained. After arrival, all mice were allowed to acclimate to the facility's laboratory conditions for 1 week prior to experimentation.<br><br>PDX models were established by the Anti-tumor Assessment Core, from tumor specimens collected under an approved institutional review board protocol by the Research Animal Resource Center and Institutional Animal Care and Use Committee at MSK, NY [reference 50 of the paper]. Briefly, tumors were minced, mixed with Matrigel, and implanted subcutaneously in 6–8-week-old NSG female mice (Jackson Laboratories). PDXs used in imaging and therapeutic experiments were obtained from patients prior initiating Trastuzumab therapy.
Wild animals	no wild animals were used in the study.
Field-collected samples	no field collected samples were used in the study.
Ethics oversight	The experimentation involving animals followed the guidelines approved by the Research Animal Resource Center and Institutional Animal Care and Use Committee at MSK (New York, NY), the ARRIVE guidelines, and the guidelines for the welfare and use of animals in cancer research.

Note that full information on the approval of the study protocol must also be provided in the manuscript.

Clinical data

Policy information about clinical studies

All manuscripts should comply with the ICMJE guidelines for publication of clinical research and a completed CONSORT checklist must be included with all submissions.

Clinical trial registration	Trastuzumab trials at MSK led by Y.J. (NCT02954536, IRB# 06-103, NCT01913639, NCT01522768).

Study protocol	N/A
Data collection	Patient clinical information was collected manually from the electronic medical record (M.M. and M.L.). The presence of somatic alterations in HER2-expressing tumors was analyzed by MSK-IMPACT.
Outcomes	HER2-positivity was defined as HER2 IHC 3+, HER2 IHC 2+ and HER2:CEP17 FISH ratio ≥ 2.0 or ERBB2-amplified by next-generation sequencing in gastric tumor tissues.

Dual use research of concern

Policy information about dual use research of concern

Hazards

Could the accidental, deliberate or reckless misuse of agents or technologies generated in the work, or the application of information presented in the manuscript, pose a threat to:

No	Yes	
☒	☐	Public health
☒	☐	National security
☒	☐	Crops and/or livestock
☒	☐	Ecosystems
☒	☐	Any other significant area

Experiments of concern

Does the work involve any of these experiments of concern:

No	Yes	
☒	☐	Demonstrate how to render a vaccine ineffective
☒	☐	Confer resistance to therapeutically useful antibiotics or antiviral agents
☒	☐	Enhance the virulence of a pathogen or render a nonpathogen virulent
☒	☐	Increase transmissibility of a pathogen
☒	☐	Alter the host range of a pathogen
☒	☐	Enable evasion of diagnostic/detection modalities
☒	☐	Enable the weaponization of a biological agent or toxin
☒	☐	Any other potentially harmful combination of experiments and agents
